# Whole-Genome Sequence of Entomortierella parvispora E1425, a Mucoromycotan Fungus Associated with *Burkholderiaceae*-Related Endosymbiotic Bacteria

**DOI:** 10.1128/mra.01101-21

**Published:** 2022-01-13

**Authors:** Afri Herlambang, Yong Guo, Yusuke Takashima, Kazuhiko Narisawa, Hiroyuki Ohta, Tomoyasu Nishizawa

**Affiliations:** a Graduate School of Agriculture, Ibaraki University, Ami, Ibaraki, Japan; b College of Agriculture, Ibaraki University, Ami, Ibaraki, Japan; c Sugadaira Research Station, Mountain Science Center, University of Tsukuba, Ueda, Nagano, Japan; d Ibaraki University, Mito, Ibaraki, Japan; Vanderbilt University

## Abstract

Some mucoromycotan fungi establish symbiotic associations with endohyphal bacteria. Here, the genome of Entomortierella parvispora E1425 (synonymously known as Mortierella parvispora E1425), which harbors a cultured *Burkholderiaceae*-related endobacterium (BRE) designated *Mycoavidus* sp. strain B2-EB, was sequenced. We provide genomic information to elucidate fungal-BRE symbiotic features.

## ANNOUNCEMENT

Soilborne fungi of *Mortierella* spp. harbor *Burkholderiaceae*-related endobacteria (BRE) (1–7) that can protect the host from nematode attack and inhibit zygospore formation (8, 9). Entomortierella parvispora E1425 (JCM 39028, synonymously known as *Mortierella parvispora* E1425) isolated from forest soil possesses a cultivable BRE named *Mycoavidus* sp. strain B2-EB (JCM 33615) (6, 10). Here, we conducted whole-genome sequencing of the host fungus.

To eliminate the endobacteria, germinated sporangiospores of E1425 were treated with ciprofloxacin (50 μg mL^−1^) at 23°C for 24 h and then incubated on fresh _LC_A medium (6) until fungal colonies appeared (9). After confirming the absence of BRE by diagnostic PCR in accordance with Takashima et al. (9), an endobacterium-cured line of E1425 was established and incubated on half-strength cornmeal-malt-yeast (CMMY) medium (2) at 23°C for 7 days (9). DNA and RNA were extracted from homogenized mycelia of endobacterium-cured E1425 using phenol-chloroform extraction (11) with a Genomic tip 100/G (Qiagen) and an RNeasy minikit (Qiagen), respectively. DNA libraries were constructed using a 1D Genomic DNA library kit (Oxford Nanopore Technologies) and a TruSeq DNA PCR-free prep kit (Illumina) and then sequenced on GridION (Oxford Nanopore Technologies) and Illumina HiSeq 2500 (2 × 150 bp) instruments, respectively. An RNA library was constructed using a TruSeq RNA library prep kit (Illumina) and sequenced on the HiSeq 2500 platform (2 × 150 bp).

Overall, 16.6-, 4.8-, and 7.4-Gbp reads were generated from the DNA-Nanopore, DNA-HiSeq, and RNA-HiSeq libraries, respectively. To determine the chromosomal genome, the DNA-Nanopore reads were processed using SeqKit (12), NanoFilt (13), and Canu (14) and then assembled using SMARTdenovo (15), followed by a polishing step using Nanopolish (16) and Pilon (17) with the cleaned DNA-HiSeq reads using Cutadapt (18). To obtain the mitochondrial genome, 5% of the cleaned DNA-Nanopore and DNA-HiSeq reads were assembled and circularized using Unicycler (19). The Cutadapt-cleaned RNA-HiSeq reads were mapped to the genome sequences using HISAT (20) to determine transcriptional sequences. The chromosomal genome was annotated using Barrnap, tRNAscan-SE (21), AUGUSTUS (22), and KofamKOALA (23), whereas the mitochondrial genome was annotated using MFannot. The software version and parameter settings are described in Table 1.

**TABLE 1:** Software version and parameter settings used for data processing

Software	Version	Process	Parameter setting(s)	Website
SeqKit	0.7.2	Quality control	seq -m 1000	https://github.com/shenwei356/seqkit
NanoFilt	2.0.0	Quality control	-q 8	https://github.com/wdecoster/nanofilt
Cutadapt	1.16	Quality control	--overlap 10, --minimum-length 51, --quality-cutoff 20	https://github.com/marcelm/cutadapt
Canu	1.7.1	Read correction	-correct, genomeSize = 40m	https://github.com/marbl/canu
SMARTdenovo	Not available	Assembly	Default settings	https://github.com/ruanjue/smartdenovo
Nanopolish	0.10.1	Genome polish	Default settings	https://github.com/jts/nanopolish
Pilon	1.22	Genome polish	Default settings	https://github.com/broadinstitute/pilon
Unicycler	0.4.7	Assembly, genome closing	Default settings	https://github.com/rrwick/Unicycler
HISAT	2.1.0	Read mapping	--rna-strandness RF, --max-introlen 10000	https://github.com/DaehwanKimLab/hisat2
Barrnap	0.9	rRNA prediction	--kingdom euk	https://github.com/tseemann/barrnap
tRNAscan-SE	2.0	tRNA prediction	Sequence source: Eukaryotic	http://lowelab.ucsc.edu/tRNAscan-SE/
AUGUSTUS	3.3.3	CDS prediction	cDNA file: the transcriptional sequences	http://bioinf.uni-greifswald.de/webaugustus/
KofamKOALA	2021-02-04	Gene annotation	Default settings	https://www.genome.jp/tools/kofamkoala/
MFannot	Not available	Mitochondrial genome annotation	Genetic code: 4	https://megasun.bch.umontreal.ca/cgi-bin/mfannot/mfannotInterface.pl

The chromosomal genome had a total size of 38,663,708 bp (coverage, ×409) in 19 contigs with a GC content of 49.4% and an *N*_50_ value of 2,802,632 bp. The circularized mitochondrial genome had a size of 66,033 bp (coverage, ×3,965) with a GC content of 24.0%. In total, 20 rRNAs, 192 tRNAs, and 11,641 coding DNA sequences (CDSs) were predicted from the chromosomal sequences, whereas 2 rRNAs, 25 tRNAs, and 29 CDSs were encoded in the mitochondrial genome. Genome annotation revealed that E1425 can synthesize various amino acids and fatty acids, which potentially served as nutrients for endobacterial growth (4, 10). Further study will be necessary to elucidate the interactions between the host fungus and BRE.

### Data availability.

The genome sequence of *Entomortierella parvispora* E1425 was deposited in the DDBJ/ENA/GenBank databases with the accession numbers BQFW01000001 to BQFW01000019 and LC659289 for the mitochondrion. The raw read data are available with the accession numbers DRA010776 and DRA013164 for the DNA sequencing (DNA-seq) and transcriptome (RNA-seq) libraries, respectively.
